# Single Nucleotide Polymorphisms as Prognostic and Predictive Factors of Adjuvant Chemotherapy in Colorectal Cancer of Stages I and II

**DOI:** 10.1155/2016/2139489

**Published:** 2016-01-14

**Authors:** Matej Horvat, Uroš Potočnik, Katja Repnik, Rajko Kavalar, Borut Štabuc

**Affiliations:** ^1^University Medical Centre Maribor, 2000 Maribor, Slovenia; ^2^Faculty of Medicine, Centre for Human Molecular Genetics and Pharmacogenomics, University of Maribor, 2000 Maribor, Slovenia; ^3^Faculty for Chemistry and Chemical Engineering, Laboratory for Biochemistry, Molecular Biology and Genomics, University of Maribor, 2000 Maribor, Slovenia; ^4^University Medical Centre Ljubljana, 1000 Ljubljana, Slovenia

## Abstract

Colorectal cancer (CRC) is a highly heterogeneous disease regarding the stage at time of diagnosis and there is special attention regarding adjuvant chemotherapy in unselected patients with stage I and stage II. The clinicohistologically based TNM staging system with emphasis on histological evaluation of primary tumor and resected regional lymph nodes remains the standard of staging, but it has restricted sensitivity resulting in false downward stage migration. Molecular characteristics might predispose tumors to a worse prognosis and identification of those enables identifying patients with high risk of disease recurrence. Suitable predictive markers also enable choosing the most appropriate therapy. The current challenge facing adjuvant chemotherapy in stages I and II CRC is choosing patients with the highest risk of disease recurrence who are going to derive most benefit without facing unnecessary adverse effects. Single nucleotide polymorphisms (SNPs) are one of the potential molecular markers that might help us identify patients with unfavorable prognostic factors regarding disease initiation and recurrence and could determine selection of an appropriate chemotherapy regimen in the adjuvant and metastatic setting. In this paper, we discuss SNPs of genes involved in the multistep processes of cancerogenesis, metastasis, and the metabolism of chemotherapy that might prove clinically significant.

## 1. Introduction

Colorectal cancer (CRC) represents the third most common malignancy worldwide in men and the second most common malignancy in women, accounting for approximately 10% of all tumor types worldwide and 8% of cancer related mortality [1]. With the advance in specific oncological treatment and earlier diagnosis due to screening programs, 5-year survival rates in CRC have risen from 56,5% for patients diagnosed in the early 1980s to 63,2% for those diagnosed in the early 1990s and most recently to 64,9% for those diagnosed after 2000 [2].

CRC is a highly heterogeneous disease regarding the stage at time of diagnosis. Approximately 15% will have node-negative, localized, stage I disease (T1-T2, N0, M0), 25% will have node-negative, locally more advanced stage II disease (T3-T4, N0, M0), 35% will have node-positive stage III disease (any T, N1-2, M0), and 25% will have advanced stage IV disease (any T, any N, M1) [3]. With surgery alone, the overall survival at 5 years for unselected patients with stage II is about 80%, with adjuvant chemotherapy offering a minimal incremental benefit in survival of less than 5%. For patients with stage III survival with surgery alone, the overall survival rate is approximately 50%. The difference in survival with all treatment modalities is highly different between stages, with 90,8% for localized disease of stages I and II, 69,5% for stage III, and 11,3% for stage IV [4].

The current treatment for resectable CRC of stages I, II, and III is surgical resection. For patients of stage I, surgical resection is the only recommended treatment without adjuvant chemotherapy. For patients of stage III, adjuvant chemotherapy is recommended for every patient. In contrast, for patients of stage II, adjuvant chemotherapy is not recommended for unselected patients [5–7]. Even though stage I and early stage II CRC are prognostically very favorable, with a small burden of disease, a proportion of these tumors have certain characteristics, making them clinically more malignant and therefore predisposing them to disease recurrence or metachronous colon cancer [8].

Single nucleotide polymorphisms (SNPs) represent a useful biomarker with the potential of being a prognostic and/or predictive factor for everyday clinical practice and decision making, but it is necessary to discover which out of nearly 150 million SNPs in the human genome have clinical significance either influencing the risk of CRC incidence, or disease dissemination, or influencing chemotherapy metabolism and thereby efficiency and side effects. We discuss in our review several SNPs that are potential prognostic factors in the adjuvant setting, because they are more frequent in metastatic disease. We also discuss several SNPs that are potential predictive factors, because of their role in chemotherapy metabolism and thereby influence on efficiency and incidence of adverse effects.

## 2. Prognostic Factors in Colorectal Cancer

There is special attention and controversy regarding stage I and stage II disease. Stage II especially is a highly heterogeneous disease, with 5-year survival of stage IIA patients being approximately 87,5% and stage IIC approximately 58,4% [9]. Out of 40% of CRC patients with pathological-negative lymph nodes by current methods of analysis (pN0), up to 30% of patients with stage I and up to 50% of patients with stage II disease will develop metastatic disease during the course of their follow-up. Furthermore, metastatic disease is present in 25% of CRC patients at the time of diagnosis and another 20–30% of patients with stages I, II, and III are going to develop metastasis after potentially curable surgical treatment, meaning that altogether up to 50% of CRC patients will eventually develop metastases [10].

Even with the advent of new chemotherapy regimens and targeted therapies, only potential curative treatment for metastatic disease is surgery in selected patients with liver metastases [11]. It is therefore important to identify patients with micrometastasis that are potentially curable. Identification of suitable prognostic markers enables clinicians to identify patients with a high risk of disease recurrence and identification of suitable predictive markers enables clinicians to choose the most appropriate adjuvant therapy [12].

Potential clinical and pathological risk factors for recurrence of stage II CRC have been investigated and incorporated in different guidelines, but a definite consensus has not yet been reached. According to European and American guidelines (The European Society for Medical Oncology (ESMO), The American Society of Clinical Oncology (ASCO), and The National Comprehensive Cancer Network (NCCN)), negative prognostic risk factors according to all three sets of guidelines are T4 tumors, bowel perforation, inadequately sampled lymph nodes, and poorly differentiated histology. Further negative prognostic markers included in one or two sets of guidelines are bowel obstruction, lymphovascular invasion, perineural invasion, and indeterminate or positive margins [5–7].

Prognostic significance as pathological risk factors in CRC may also have tumor budding, modified classical grading system, lymphocytic infiltration, and circumferential margin involvement [13–16]. Tumor budding is an independent marker of a potentially poor outcome in CRC, but there is a lack of uniformity regarding tumor budding [17]. It is defined as presence of individual tumor cells and clusters of tumor cells at the invasive front of the tumor and it has been postulated to represent an epithelial-mesenchymal transition, a critical mechanism for the acquisition of malignant phenotypes [18]. Another promising pathohistological feature is the modified classical grading system according to WHO. The grading system currently in use does not encompass rare subtypes of CRC and is subject to interobserver variability. A novel grading system was recently proposed: the poorly differentiated clusters. It is defined by counting clusters composed of 5 or more cancer cells and lacking a gland-like structure [15]. Circumferential margin involvement is especially important in rectal cancer and is a strong predictor for local recurrence after surgery when associated with a margin of less than 2 mm, but it also presents with an increased risk of distant metastases with margins of less than 1 mm [16].

Possible new prognostic and predictive factors emerging in CRC are due to immunological properties next to classical TNM classification. A worldwide task force has researched immunological characteristics of the tumor and translated them into “immunoscore.” Two lymphocyte populations (CD8 and CD45RO) were evaluated in the center of the tumor and invasive margin and graded accordingly to its density. It was discovered that patients with higher lymphocytic densities had a statistically lower relapse rate [19].

The most important prognostic marker of resectable CRC survival is still regional lymph node involvement; however, this is greatly influenced by the number of lymph nodes resected by the surgeon and by the number of lymph nodes examined by the pathologist. The clinicohistologically based TNM staging system with emphasis on histological evaluation of primary tumor and regional resected lymph nodes remains the standard of staging, but imprecision reflects its limitations. Microscopy has restricted sensitivity with normal detection limits of one cancer cell in about 200 [20]. Histology typically reviews less than 0,1% of biopsied tissue producing a sampling error, as more than 99,9% of available tissue is not examined. Another limitation is inhomogeneous distribution of cancer cells in biopsied tissue, which may produce a substantial amount of false-negative results regarding regional lymph node status resulting in false-negative downward stage migration. These inadequacies can be overcome by molecular staging with molecular detection of occult lymph node metastasis producing an independent indicator of prognostic risk of CRC recurrence [21].

## 3. Adjuvant Chemotherapy in Colorectal Cancer

Adjuvant chemotherapy is the standard treatment in stage III CRC being introduced into everyday clinical practice in the early 1990s, first as a combination of 5-FU and levamisole administered for 12 months and later 6 months of therapy with the same survival benefit [22]. Oral capecitabine has also proven to be equivalent to intravenous 5-FU plus leucovorin [23]. In 2004, oxaliplatin in combination with 5-FU was proven to be superior to 5-FU/LV and was approved as a standard regimen for adjuvant chemotherapy in stage III CRC with an absolute benefit of 8% to 10% [24, 25]. Also, the combination of oxaliplatin with capecitabine, a 5-FU prodrug, has also proven to be superior to 5-FU/LV and equivalent to a combination of oxaliplatin and 5-FU in terms of efficiency [26]. Systemic treatment of CRC in the metastatic setting has been extensively modified in the last decade, but in the adjuvant setting only 5-FU and its prodrug capecitabine either as monotherapy or in combination with oxaliplatin have a statistically significant effect on overall survival. No benefit of targeted therapy or irinotecan in unselected patients has been observed [27, 28].

For stage I CRC, according to clinical practice guidelines, wide surgical resection with formation of anastomosis is the treatment of choice. Adjuvant chemotherapy is not required [5–7].

Adjuvant chemotherapy in stage II remains much more controversial. In the QUASAR study, chemotherapy with 5-FU and oxaliplatin improved survival in unselected stage II patients by merely 3,6%. Also the number of resected lymph nodes was inadequate (less than 12) in a proportion of patients, making it probable that the stage was underestimated [29]. Other pooled retrospective analyses also showed conflicting results. One analysis suggested an approximate 8% absolute reduction in mortality, whereas a similar analysis showed no benefit from adjuvant chemotherapy [30, 31]. A meta-analysis published in 2012 showed a benefit with adjuvant chemotherapy in stage II colon cancer patients, but the trials used various chemotherapy regimens. There was also lack of surgical quality control and the reported differences were small [32]. There is also the question of adding oxaliplatin to fluoropyrimidine backbone with stage II CRC patients. Results from the C-07 trials did not show a benefit of adding oxaliplatin in adjuvant chemotherapy for stage II patients regarding overall survival, but a hint of benefit regarding disease free survival [33]. It remains uncertain whether patients with stage II CRC derive sufficient benefit from adjuvant chemotherapy [34]. Adjuvant treatment is recommended for all patients with stage III disease and taken altogether stage IIIA disease (T1-2, N1) appears to be associated with statistically significantly improved survival compared with that of stage IIC disease [35]. Adjuvant chemotherapy in stage II CRC is recommended for selected patients with clinical or pathological risk factors. The problem is that these risk factors differ according to different clinical treatment guidelines. Consensus on them has not been reached yet. There is no clear message regarding adjuvant chemotherapy patient selection in stage II CRC [5–7].

Regional lymph node status regarding infiltration with cancer cells is at the moment the most important prognostic factor in several solid tumors, but patients with node-negative disease are still at a significant risk of disease recurrence and development of distant metastasis making the development of a more accurate staging system based on either molecular or immunological characteristics of patient and tumor of paramount importance [36]. Histologically identical tumors may have a drastically different prognosis and/or response to treatment and these discoveries prompted the theory that, rather than a single malignancy, CRC is a heterogeneous, multifactorial disease and that individual tumors are initiated and progress in a unique manner, which is not necessarily identical amongst all tumors. As a result of this, CRC research is shifting from a clinical and pathohistological perspective towards developing an understanding of the molecular basis of this malignancy, including individual susceptibility, development, progression response, and resistance to antitumor treatment and metastatic spread [37].

## 4. Molecular Prognostic Factors in Resectable Colorectal Cancer

Patients and physicians are on a daily basis confronted with choices about whether to embark on a course of adjuvant chemotherapy designed to eliminate micrometastatic disease after potentially curative surgical treatment, or whether not to administer treatment and which treatment to choose. In elderly patients aged more than 70 years and in patients with comorbidities, the importance of choosing an appropriate regimen with acceptable toxicity is of special importance, as these patients may not be as robust as their younger counterparts and patients with no comorbidities and because they may place a different value on the time and logistical challenges relevant to a course of a treatment [38].

Molecular mechanisms regulating cancer progression in solid tumors encompass genomic, transcriptome, and epigenetic alterations. The latter significantly contribute to tumor progression particularly in early stage metastasis, in which acquisition of epigenetic alterations of involvement of epigenetic factors may allow cancer cells to disseminate from the primary tumor and metastasize to and survive at a distant site [39]. Growing evidence supports the idea that epithelial cancers including CRC are diseases driven by pluripotent, self-renewing cancer stem cells, which are chemotherapy resistant, and that this is one of the primary causes for tumor recurrence [40].

The most prominent molecular predictive marker in CRC is the KRAS mutation identifying 40% of patients that will not respond to anti-EGFR treatment with cetuximab or panitumumab. Nonetheless, it has no significance in the adjuvant setting as targeted therapies did not show any clinical benefit [41]. A molecular characteristic important in the adjuvant setting is microsatellite instability (MSI-H), being a favorable prognostic factor in all disease stages in comparison to microsatellite stable tumors (MSS) [42]. Besides being a favorable prognostic marker, it also seems to predispose chemoresistance to 5-FU-based chemotherapy, thereby representing a negative predictive marker in the adjuvant CRC setting [43]. Both of these facts make MSI-H patients less suitable candidates for adjuvant chemotherapy. The recent ESMO clinical practice guidelines suggest testing for MSI-H in stage II colon cancer in order to contribute in treatment decision making regarding chemotherapy administration [44]. The role of oxaliplatin-based therapy is still being evaluated in the MSI-H setting [45]. There might even be a role for irinotecan-based therapy in MSI-H patients in the adjuvant setting [46].

High penetrance mutations, such as those of APC and MMR genes, account for less than 5% of cases in the pathogenesis of CRC [47, 48]. Another 8,3% of cases arise in families with two affected first- or second-degree relatives with mildly or moderately penetrating alleles explaining the familial aggregation. The remaining proportion of inherited susceptibility that is as high as −35% as discovered by twin studies is likely to be explained by low-risk variants that can also depend on several parameters in any given population (mutation rate, genetic selection, and population demography) thereby conferring risk in one population, but not in another [49, 50].

## 5. Single Nucleotide Polymorphisms in Resectable Colorectal Cancer

Genome-wide association studies (GWAS) enable us to examine many common genetic variants in different individuals thereby discovering if any variant is associated with a trait as predisposition to developing cancer. SNP is a DNA sequence variation occurring within a population with an inhomogeneous distribution, occurring more frequently in noncoding regions with other factors such as genetic recombination and mutation rate determining SNP density [51].

There are two different types of SNPs influencing incidence of diseases. If the variation of the SNP is located within or close to the translated region, any amino acid substitutions that alter protein synthesis could be directly connected to the disease with a straightforward correlation. On the other hand, if a disease-associated SNP is located in a noncoding region or there is no gene near the SNP, then it is more difficult to determine the mechanism by which the SNP is associated with the disease [52]. A significant factor of heterogenous distribution is microsatellites, in particular, AT microsatellites being a potent predictor of reduced SNP density [53]. The GWAS approach has proved powerful in identifying robust associations between many SNPs and traits, but additional work is needed to determine the functional basis for the observed associations [54].

Several studies regarding CRC incidence have been conducted in the last decade, but our special interest is on studies regarding germline SNPs that may prove to be important in the CRC adjuvant chemotherapy setting. SNPs are characteristics of genes that may in certain cases be regarded as negative prognostic factors and may predispose patients to disease recurrence.

### 5.1. Single Nucleotide Polymorphisms as a Predictive Marker

SNPs can be predictive markers in CRC therapy, because of their role in genetic pathways involved in metabolism, cellular transport, and the mechanisms of action of chemotherapy agents. They can thereby influence response to treatment [55]. As aforementioned, the standard adjuvant chemotherapy is 5-FU or its prodrug capecitabine as monotherapy or a combination of 5-FU or capecitabine with oxaliplatin (FOLFOX or XELOX regimen). Identical chemotherapy regimen is one of the standard chemotherapy regimens in metastatic CRC [56].

Several pharmacogenetic studies were conducted regarding response to treatment with oxaliplatin and 5-FU. Polymorphisms* ERCC2* Lys751Gln and* XRCC1* Arg399Gln showed worse response and shorter survival to chemotherapy. Both studies were conducted in the advanced CRC setting with either FOLFOX or XELOX regimen [57, 58]. Ye et al. [59] conducted a meta-analysis concerning* XRCC1* and* GSTP1* polymorphisms and prognosis with oxaliplatin-based chemotherapy in metastatic CRC.* XRCC1* Arg399Gln polymorphism was significantly associated with oxaliplatin-based chemotherapy response in CRC when stable disease and disease progression were defined as nonresponse, although the association was not significant when only disease progression was considered as nonresponse. The tumor response rate was significantly lower in patients who carried Arg/Gln or Gln/Gln genotypes. The present meta-analysis did not show a significant relationship between tumor response and* GSTP1* polymorphisms. In clinical studies performed in advanced colorectal setting with oxaliplatin/5-FU-based chemotherapy regimens, polymorphisms* GSTP1* Ile105Val,* MGMT −*535G/T,* MTHFR* 677C/T, and* MTHFR* 1298A/C showed longer time to progression or longer survival [60–63]. Studies performed in the adjuvant CRC setting have also discovered a predictive role of SNPs. Cecchin et al. [64] conducted a study concerning* MTHFR* 1298A>C (rs1801131) polymorphism as a predictor of survival in two cohorts of stage II/III setting of CRC patients treated with adjuvant fluoropyrimidine chemotherapy with or without oxaliplatin.* MTHFR* 1298CC genotype carriers had worse disease free survival and also worse overall survival in both cohorts. They concluded* MTHFR* 1298A>C is a prognostic factor that could be an additional criterion for the choice of a proper adjuvant regimen in the adjuvant setting. Kap et al. [65] conducted a study concerning genetic variants in the glutathione S-transferase (*GST*) genes and survival in CRC patients after chemotherapy and differences according to treatment with oxaliplatin. Patients with stages II–IV were included. CRC patients who were homozygote carriers of* GSTM1* had significantly poorer survival after treatment with oxaliplatin than those not treated with oxaliplatin. The association was significant in metastatic CRC patients treated with oxaliplatin. Neither the* GSTP1* 105Val allele nor the* GSTT1* deletion was significantly associated with CRC survival. These data suggest that* GSTM1* may be a predictive marker for oxaliplatin therapy. Absenger et al. [66] conducted a study concerning cyclin D1 (*CCND1*) rs9344 G>A polymorphism predicting clinical outcome in colon cancer patients treated with adjuvant 5-FU-based chemotherapy in stage II/III. Patients treated with 5-FU-based chemotherapy carrying the* CCND1* rs9344 A/A genotype had significantly decreased time-to-tumor recurrence. In the validation set, the A allele of* CCND1* SNP rs9344 remained significantly associated with decreased time-to-tumor recurrence. They concluded that* CCND1* rs9344 may be a predictive and/or prognostic biomarker in stage II/III colon cancer patients. Páez et al. [67] conducted a study concerning association of common gene variants in the Wnt/*β*-catenin pathway in colon cancer recurrence. They investigated germline polymorphisms in a panel of Wnt/*β*-catenin pathway genes to predict time-to-tumor recurrence in patients with high risk stage II and stage III. They discovered that the minor allele of* WNT5B* SNP rs2010851 was significantly associated with shorter time-to-tumor recurrence in high risk stage II patients.

Another factor in addition to treatment response is evaluating the effect of SNPs on chemotherapy toxicity. Chemotherapy adverse effects are neither stage nor disease specific. Chemotherapy regimens used in different cancers produce similar adverse effects. As well as being a predictive factor of treatment efficiency, SNPs are also predictive factors of adverse effect incidence. Caronia et al. [68] discovered an association of increased risk of hand-foot syndrome appearance with SNP rs532545 in the* CDA* gene in CRC and breast cancer patients either in the adjuvant or metastatic setting. Gusella et al. [69] discovered that* MTHFR* C677T polymorphism was protective against grade 3 and 4 toxicity in CRC patients of B2 and C Dukes stages receiving adjuvant chemotherapy with 5-FU. Argyriou et al. [70] discovered an association regarding voltage-gated sodium channel polymorphisms in the development of oxaliplatin-induced peripheral neurotoxicity in CRC patients in either the adjuvant or metastatic setting. SNPs* SCN4A*-rs2302237 and* SCN10A*-rs1263292 emerged as being significantly associated with an increased incidence of acute oxaliplatin-induced peripheral neuropathy.* SCN4A*-rs2302237 emerged also as being predictive of the clinical severity of acute oxaliplatin-induced peripheral neuropathy and the occurrence of cumulative/chronic oxaliplatin-induced peripheral neuropathy. Custodio et al. [71] discovered that* cyclin H (CCNH)* rs2230641 C/C and the ATP-binding cassette subfamily G, member 2* (ABCG2)* rs3114018 A/A, were associated with a higher risk of severe oxaliplatin-induced peripheral neuropathy in patients with stage II and III CRC. Patients harboring the combination of both genotypes had a higher risk of grades 2-3 oxaliplatin-induced peripheral neuropathy.

### 5.2. Single Nucleotide Polymorphisms as a Prognostic Marker

Metastasis is a multistep process with many genes regulating escape from the primary colorectal tumor, intravasation into the lymphatic or vascular systems, survival in circulation, avoidance of host defense mechanisms, arrest at a new site, extravasation into the tissue, and growth at the new site [72]. Among the genes involved are* SDF-1α* (stromal derived factor-1 alpha) located on chromosome 10 [73],* MMP* and* TIMP* (matrix metalloproteinases and tissue inhibitor of matrix metalloproteinases), especially* MMP-7* located on chromosome 11,* MMP-9* located on chromosome 16, and* TIMP-2* located on chromosome 17 [74–76],* RAD18* located on chromosome 3 [77], and* MACC1* (metastasis associated in colorectal cancer 1) located on chromosome 7 [78]. With research, so far, we were able to determine many processes, but the exact molecular pathways need further elucidation. SNPs present a field of ongoing research regarding the incidence and clinical manifestation of CRC and their role as a prognostic and also a predictive factor.

The SDF-1*α*/CXCR4 axis was initially found to be stimulated by the homing of lymphocytes to inflammatory tissues and has been found to be involved in many areas of immunology and human development, including organogenesis, vascularization, hematopoiesis, and embryogenesis [79]. The SDF-1*α*/CXCR4 axis promotes metastasis in numerous cancers. SDF-1*α* is produced and released from tissues such as liver or lung and triggers the migration of tumor cells expressing the* CXCR4* receptor thereby promoting invasion, proliferation, and survival under suboptimal condition [80]. Microenvironment conditions such as hypoxia induce* CXCR4* expression which further sensitizes tumor cells to signals such as CXCL12 and promotes tumor metastasis. All these factors show a strong correlation between* SDF-1α/CXCR4* gene expression and worse prognosis [81]. SNPs of the* SDF-1α* gene have also been studied as a factor of an increased likelihood of developing cancer. SDF-1*α* G801A polymorphism in the untranslated 3′ region was associated with increased likelihood of developing breast and lung cancer [82, 83]. It also increases the likelihood of dissemination of breast cancer and leukemia [84, 85]. Chang et al. [86] conducted a clinical trial regarding the frequency of six SNPs of the* SDF-1α* gene in patients with T3 colon cancer with and without lymph node metastasis. Among the six SNPs, the frequency of GA/AA genotype of G801A (G12197A, rs1801157) was significantly higher in patients with lymph node metastasis than in those without metastasis. In addition, an investigation of the relationship between* SDF-1α* genotypes and different clinicopathological prognostic factors revealed a positive association between the GA/AA genotype and lymphovascular invasion. Systemic dissemination and the frequency of* SDF-1α* polymorphisms were not evaluated. Based on these results, a novel therapeutic strategy might be inhibition of the activated SDF-1*α*/CXCR4 signal pathway.

*MACC1* is a regulator of the HGF/Met signaling pathway and plays a key role in regulating many biological processes including cellular proliferation, cell metastasis, cell invasiveness, and angiogenesis. Furthermore, the activation of the HGF/Met signaling pathway was also a key step to epithelial-mesenchymal transition, inducing increased invasiveness, tumorigenesis, and also chemoresistance [87]. The* MACC1* gene was discovered by a genome-wide search in human colon cancer tissues, metastases, and normal tissues. High expression levels of* MACC1* correlate positively with colon cancer metastases and reduced metastasis-free survival [78]. It has been shown that overexpression of* MACC1* correlates better with unfavorable pathologic features than overexpression of* MET* [88]. Studies indicate that* MACC1* may also involve other signaling pathways such as AKT and Ras/ERK. It may also affect levels of MMP-2 and MMP-9 [89].* MACC1* promotes the carcinogenesis of CRC and is associated with the transition from adenoma to carcinoma and the invasive growth of early CRC [90].* MACC1, HGF,* and* MET* are all located on chromosome 7 and amongst their neighbors are genes that are known to be involved in signal transduction and regulation of cell adhesion and motility. For instance,* TWIST* as well as* ITGB8* is known to contribute to the tumorigenesis and metastasis of CRC [91]. Expression levels of* MACC1* in colon cancer without distant metastases were significantly higher in primary tumors that later developed distant metastases, compared to those that did not metastasize within a 10-year follow-up period. Thus,* MACC1* represents an early prognostic indicator for colon cancer metastases that is independent of age, sex, tumor infiltration, nodal status, and lymph vessel invasion [92]. Several SNPs have been discovered in the human* MACC1* gene and Lang et al. conducted a trial researching six SNPs in* MACC1* gene from formalin-fixed paraffin-embedded CRC tissue. They reported a positive association of the* MACC1* tagging SNP rs1990172 located in the intron region of a gene with reduced overall survival in patients with CRC. Remaining SNPs of the* MACC1* locus did not show a significant impact on overall survival [93]. Schmid et al. [94] conducted a trial on SNPs in the coding region of* MACC1* and the clinical outcome of CRC and discovered that SNP might be associated with a reduced survival for younger colon cancer patients in early stages.

The genetic polymorphisms of DNA repair genes were analyzed to determine the susceptibility to several cancers including lung, head and neck, breast, bladder, leukemia, and also colorectal cancer. The* RAD18* gene combines two distinct pathways maintaining genome stability. On the one hand, RAD18 acts with E2 conjugating enzyme RAD6 to promote PCNA monoubiquitination at stalled replication forks, which initiate the DNA damage bypass pathway. On the other hand,* RAD18* can also transmit DNA damage signaling to elicit homologous recombination repair after DNA damage, via a well-defined DNA damage signaling pathway [95]. DNA lesions induced by mutagens, including UV light and chemicals, are thereby efficiently removed. RAD18 recruits RAD6 to the site of DNA damage and the complex binds to damaged DNA by the single-stranded via DNA-binding activity of RAD18, where RAD6 then modulates stalled DNA replication through their ubiquitin-conjugating activity [96]. Kanzaki et al. [77] conducted a study in Japan regarding correlation of SNPs in the* RAD18* gene and risk of CRC and they discovered that SNP Arg302Gln is associated with increased risk of CRC. They also found a significant association between polymorphism and clinicopathological features, specifically in differentiated grade and lymph node metastasis. Pan et al. [97] conducted a study in China regarding SNPs in* TLS* (translesion synthesis polymerases) and susceptibility and metastasis in CRC. They discovered that SNPs in two* TLS* genes increased the risk of developing CRC and that polymorphism rs373572 in* RAD18* is significantly related to an increased risk of metastasis in CRC patients.

MMPs form a family of zinc dependent endopeptidases that degrade the extracellular matrix, but also nonmatrix, proteins. There are 24 known human MMPs, and they are classified based partly on their substrate specificity and partly on their cellular localization as follows:* collagenases, gelatinases, stromelysins, matrilysins, membrane-type MMP,* and others [98]. Function of MMPs is regulated by another group of enzymes named* TIMP*. This group encompasses four TIMPs:* TIMP-1, TIMP-2, TIMP-3, and TIMP-4* [99]. The MMPs and TIMPs play a key role in the normal physiology of connective tissue during development, morphogenesis, and wound healing, but their unregulated activity has been implicated in numerous disease processes including arthritis, atherosclerosis, and tumor cell metastasis [100]. It has been proposed that MMPs and TIMPs might play a role not only in tumor invasion and initiation of metastasis, but also in carcinogenesis from colorectal adenomas, and several studies demonstrated that high preoperative serum or plasma MMPs and TIMPs antigen levels are strong predictive factors for poor prognosis in patients with CRC [101]. The overexpression of MMP-7, MMP-9, and TIMP-2 indicates they have considerable metastatic potential and correlates with unfavorable clinicopathological characteristics [102, 103]. Further studies discovered the impact of SNPs in genes* MMP-7, MMP-9,* and* TIMP-2* in CRC tumor progression and metastasis. Park et al. [104] conducted a study in Korea regarding the impact of SNPs in genes* TIMP-2, MMP-2, and MMP-9* on clinical characteristics in CRC. The study demonstrated that SNPs in* TIMP-2* are associated with CRC susceptibility and pathological characteristics. Of special interest is the fact that the frequency of* TIMP-2* rs81799090 genotype G/G was higher in patients with metastasis than in those without metastasis. Dziki et al. [105] conducted a study in Poland regarding rs11568818 polymorphism of the* MMP-7* gene promoter region in CRC. They discovered a possible relationship between functional SNP and the susceptibility to development of CRC and an aggressive course of the disease. Xing et al. [106] conducted a study to explore the role of the* MMP-9* polymorphism in CRC. Their results indicate that the* MMP-9* 1562C>T rs3918242 polymorphism located within an important regulatory element that appears to be a binding site for a transcription repressor protein affects lymph node metastasis in CRC.

## 6. Conclusion

The search for new prognostic factors, either new biomarkers or modified pathohistological characteristics, is a never-ending story and it will be the subject of further research. A personalized approach in medicine has in the last decade changed the field of oncology. The clinical and pathological TNM staging system is still the most important prognostic factor, but in certain cancers and stages due to heterogeneous disease, it produces limited prognostic and no predictive information.

Novel molecular targets and therapeutic agents have led to the study of biomarkers as prognostic and predictive factors. Understanding the molecular mechanisms underlying the carcinogenesis and metastatic process will help us to identify those at the highest risk of recurrence and to find new tumor targets to prevent disease progression. One of the novel biomarkers in CRC might be SNPs that influence disease incidence and have the potential of becoming a prognostic and/or predictive factor for everyday clinical practice and decision making; however, it is important to identify those SNPs with the strongest predisposition.

Surgery is still the mainstay of CRC treatment, but chemotherapy further lowers recurrence rate and improves survival. It is important to treat patients with resectable CRC as effectively as possible, choosing patients with the highest probability of recurrence that benefit the most from adjuvant treatment and not to overtreat patients with low probability of disease recurrence. More intensive follow-up strategies might also be applied in patients with higher recurrence probability. Furthermore, targeted strategies against these aforementioned genes might prove to be a reasonable therapeutic option.

The potential characteristics of SNPs have special significance in that they may enable us to discover patients with higher risks of disease recurrence of stage I and stage II. In addition, the potential effect of SNPs on chemotherapy metabolism may also be of great benefit. Individualized decision making is recommended given the unclear benefit of adjuvant chemotherapy in unselected patients of stages I and II. Therefore, it would be useful to determine SNPs of genes involved in the metastatic process of CRC and chemotherapy metabolism and to test their clinical significance.

Due to effectiveness, side effects, and costs, there is further need for patient-tailored therapy in the adjuvant setting, as we have in the metastatic setting with KRAS testing and choice of appropriate targeted therapy. Studies are being conducted on biomarkers such as SNPs from tumor tissue, lymph nodes, and peripheral blood, in order to determine molecular factors for patient and selection. Promising results have been discovered, but most of them need further validation in larger prospective clinical trials.
